# PPARγ activation rescues oxidative stress-induced embryonic arrest by suppressing Wnt/β-catenin signaling via GSK3β upregulation

**DOI:** 10.1016/j.isci.2026.114870

**Published:** 2026-01-30

**Authors:** Lihong Liu, Siyao Ha, Hui Chen, MingQing Li, Zhiling Li

**Affiliations:** 1 Reproductive Center, The First Affiliated Hospital of Shantou University Medical College, Shantou University, Shantou 515041, China; 2 Department of Obstetrics and Gynecology, the Reproductive Medicine Center, Sun Yat-sen Memorial Hospital, Sun Yat-sen University, Guangzhou 510120, China; 3 Guangdong Provincial Clinical Research Center for Obstetrical and Gynecological Diseases, Guangzhou 510120, China; 4 Department of Reproductive Immunology, The International Peace Maternity and Child Health Hospital, School of Medicine, Shanghai Jiao Tong University, Shanghai 200030, China; 5 Shanghai Key Laboratory of Embryo Original Diseases, Shanghai 200030, China

**Keywords:** Biological sciences, Developmental genetics, Developmental biology

## Abstract

Excessive reactive oxygen species (ROS) during assisted reproductive technology (ART) impairs embryonic development, yet the intrinsic molecular mechanisms remain inadequately understood. Through transcriptomic profiling (Drug-seq) of oxidatively stressed mouse embryos, we identified peroxisome proliferator-activated receptor gamma (PPARγ) as a critical regulator whose essential upregulation during zygotic genome activation (ZGA) is suppressed. Functional studies demonstrated that the pharmacological activation of PPARγ via the agonist GW1929 robustly rescued developmental arrest by scavenging ROS, restoring mitochondrial function, and maintaining metabolic homeostasis. Mechanistically, we demonstrate that PPARγ activation transcriptionally upregulates GSK3β, which in turn suppresses oxidative stress-induced aberrant Wnt/β-catenin signaling. Our findings establish PPARγ as a central guardian of embryonic redox and metabolic homeostasis, and propose PPARγ agonism as a potential strategy to improve ART outcomes by counteracting oxidative injury.

## Highlights

• PPARγ activation rescues oxidative stress-induced embryonic arrest
• PPARγ upregulates GSK3β to suppress aberrant Wnt/β-catenin signaling
• PPARγ agonist GW1929 restores redox/metabolic homeostasis in embryos

## Graphical abstract

**Figure:**
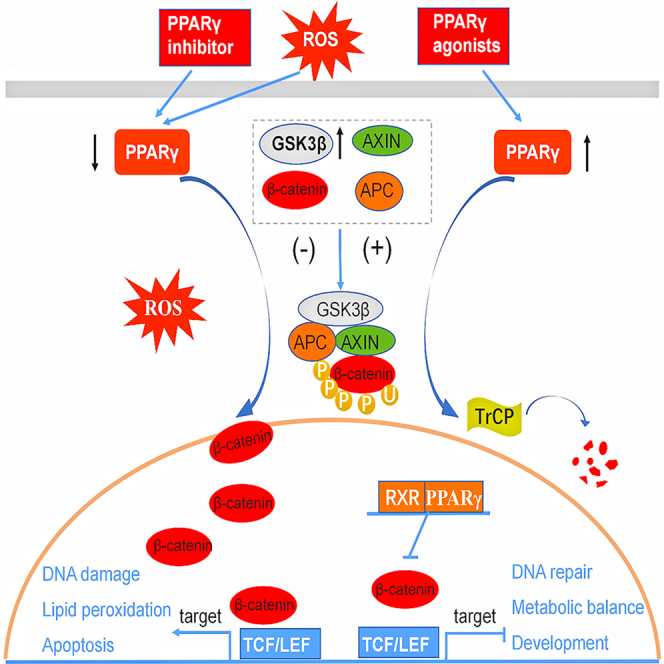

## Introduction

Assisted reproductive technology (ART) has revolutionized the treatment of infertility. However, its efficiency remains suboptimal, as evidenced by lower-than-expected blastocyst formation rates.^1^^,^^2^ A major contributing factor is the exposure of gametes and embryos to non-physiological *in vitro* culture conditions, which can induce excessive reactive oxygen species (ROS) and cause oxidative stress.^3^ Preimplantation embryos are particularly vulnerable to such oxidative damage, which disrupts metabolic homeostasis, impairs mitochondrial function, and leads to developmental arrest.^4^^,^^5^

The Wnt/β-catenin signaling pathway is a critical regulator of embryonic development and metabolism.^6^ Previous studies, including our own, have shown that oxidative stress can aberrantly activate Wnt/β-catenin signaling, resulting in DNA damage and potential long-term developmental defects.^7^^,^^8^ Conversely, the nuclear receptor PPARγ, a master regulator of lipid and glucose metabolism, is expressed throughout preimplantation development and is essential for blastocyst formation.^9^^,^^10^ Interestingly, PPARγ and Wnt/β-catenin often exhibit an antagonistic relationship in various biological contexts. PPARγ activation confers antioxidant effects in somatic cells,^11^^,^^12^ and can inhibit Wnt/β-catenin signaling via the upregulation of GSK3β, a key kinase in the β-catenin destruction complex.^13^^,^^14^

Despite these advances, the role of PPARγ as an endogenous regulator in defending against oxidative stress in preimplantation embryos and its potential crosstalk with the Wnt/β-catenin pathway remain unexplored. We therefore hypothesized that oxidative stress impairs preimplantation embryonic development by suppressing PPARγ activity, which in turn unleashes aberrant Wnt/β-catenin signaling due to compromised GSK3β-mediated inhibition.

Building on our previously established mouse zygote model of oxidative stress^15^ and subsequent finding that oxidative damage induces ribosomal biogenesis activation contributing to transgenerational tumor susceptibility through Wnt and TGF-β1 signaling pathways.^7^ Building on this foundation, we employed our oxidative damage model in combination with transcriptomic profiling, which identified that PPARγ fails to be activated during 2 cell late (ZGA) under oxidative stress. Using pharmacological interventions, we demonstrate that PPARγ activation rescues embryonic development by mitigating oxidative damage and sustaining metabolic homeostasis. Furthermore, we mechanistically unveil a novel PPARγ-GSK3β-β-catenin signaling axis that preserves genomic integrity and pluripotency. Our findings elucidate a fundamental protective mechanism and suggest PPARγ agonism as a potential therapeutic strategy to improve ART outcomes.

## Results

### Transcriptomic alterations during zygotic genome activation in oxidatively damaged zygotes

**Figure 1:**
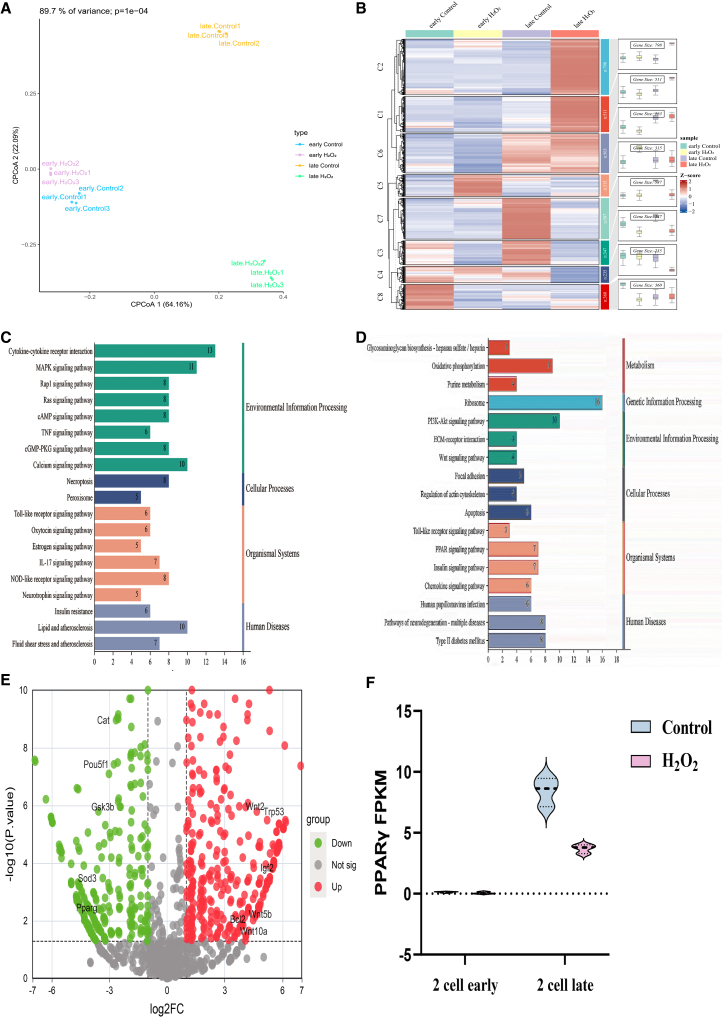
Transcriptomic profiling identifies PPARγ as a key suppressed regulator during ZGA under oxidative stress (A) Principal coordinates analysis (PCoA) of transcriptomes from control and H_2_O_2_-treated embryos at early and late two-cell stages. (B) Heatmap of DEGs across the four experimental groups. (C) KEGG pathway enrichment analysis of DEGs between control and. H_2_O_2_-treated groups at the early two-cell stage. (D) KEGG pathway analysis of DEGs at the late two-cell stage. (E) Volcano plot of DEGs between control and H_2_O_2_-treated embryos at the late two-cell stage. Significantly upregulated and downregulated genes are marked in red and green, respectively. (F) Expression levels (FPKM) of PPARγ in control and H_2_O_2_-treated embryos at early and late two-cell stages. Data from 3 independent biological replicates, n > 200 per sample.

To comprehensively map the transcriptional alterations induced by oxidative stress during the critical window of ZGA, we performed genome-wide Drug-seq analysis on embryos at the early and late 2-cell stages. Principal coordinates analysis (PCoA) revealed distinct clustering of transcriptomic profiles between control and H_2_O_2_-treated groups at the late 2-cell stage (Figure 1A). Consistent with this, a heatmap of differentially expressed genes (DEGs) demonstrated more pronounced alterations at the late 2-cell stage (Figure 1B). KEGG pathway enrichment analysis indicated that DEGs at the early 2-cell stage were primarily involved in signal transduction and metabolic pathways. In contrast, DEGs at the late 2-cell stage were associated with metabolic processes and apoptosis-related pathways, including WNT and insulin signaling pathways (Figures 1C and 1D). Volcano plot analysis showed significant downregulation of genes critical for embryonic development (e.g., OCT4), antioxidant defense (SOD3, CAT), metabolism (PPARγ, GSK3β), and apoptosis (TP53, BCL2), alongside upregulation of Igf2 (Figure 1E). Notably, while control embryos exhibited significant upregulation of PPARγ from the early to late 2-cell stage, this developmental activation was markedly abrogated by H_2_O_2_ treatment (Figure 1F). These results suggest that the failure to upregulate PPARγ during ZGA may be a key event in oxidative stress-induced developmental impairment. Based on this foundation, we next validated its expression and functional role.

### Peroxisome proliferator-activated receptor gamma expression is impaired in oxidatively damaged embryos

**Figure 2:**
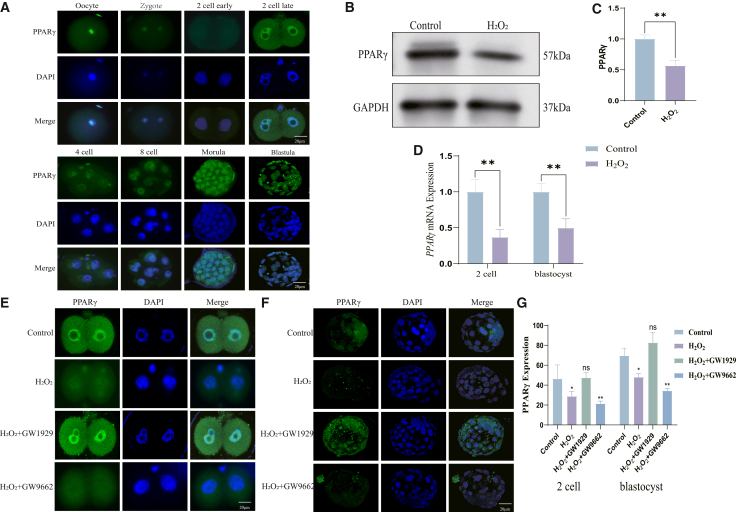
Oxidative stress impairs the expression and nuclear localization of PPARγ during early embryonic development (A) Representative immunofluorescence images showing the expression and nuclear localization (green) of PPARγ at different preimplantation stages (MII oocyte to blastocyst). Nuclei are counterstained with DAPI (blue). (B) Western blot analysis of PPARγ protein levels in 2 cell layers from control and H_2_O_2_-treated groups. (C) Quantification of PPARγ protein levels from (B). (D) qRT-PCR analysis of PPARγ mRNA levels at the 2-cell and blastocyst stages. (E) PPARγ expression in the late 2-cell stage following H_2_O_2_ treatment and subsequent culture with GW1929 or GW9662. (F) PPARγ expression in blastocyst stage following H_2_O_2_-treated and subsequent culture with GW1929 or GW9662. (G) Quantitative analysis of PPARγ expression from experiments in (E) and (F). Data are represented as mean ± SEM; *n* >300 per sample for western blot, n >5 per sample for immunofluorescence, n >300 per sample for PCR). All data from 3 independent biological replicates. ns *p* ≥ 0.05, ∗*p* < 0.05, ∗∗ *p* < 0.01, ∗∗∗ *p* < 0.001 by Student’s *t* test (or one-way ANOVA with post-hoc test). Scale bar = 20 μm.

Analysis of PPARγ expression during preimplantation development revealed a dynamic expression pattern: Immunofluorescence localization analysis revealed that PPARγ is predominantly localized to the nucleus. PPARγ is highly expressed in mature oocytes, decreases progressively after fertilization, reaches its lowest level at the early 2-cell stage, and is subsequently re-upregulated from the late 2-cell stage onward, maintaining elevated expression through the blastocyst stage (Figure 2A). Western blot analysis revealed a significant downregulation of PPARγ expression in 2-cell late stage following oxidative damage induced in zygotes (Figures 2B and 2C). This downregulation was further confirmed by qRT-PCR, which showed a consistent reduction in PPARγ mRNA levels at the 2-cell and blastocyst stages after H_2_O_2_ exposure (Figure 2D). These results establish that oxidative stress specifically impairs PPARγ expression during early embryonic development. To this end, we employed the PPARγ agonist GW1929 and antagonist GW9662 to respectively enhance or inhibit its signaling pathway in oxidatively stressed embryos (Figures 2E–2G). Given the correlation between PPARγ downregulation and developmental arrest, we next asked whether modulating PPARγ activity could functionally rescue embryonic development.

### Pharmacological activation of peroxisome proliferator-activated receptor gamma rescues embryonic development and mitigates oxidative damage

**Figure 3:**
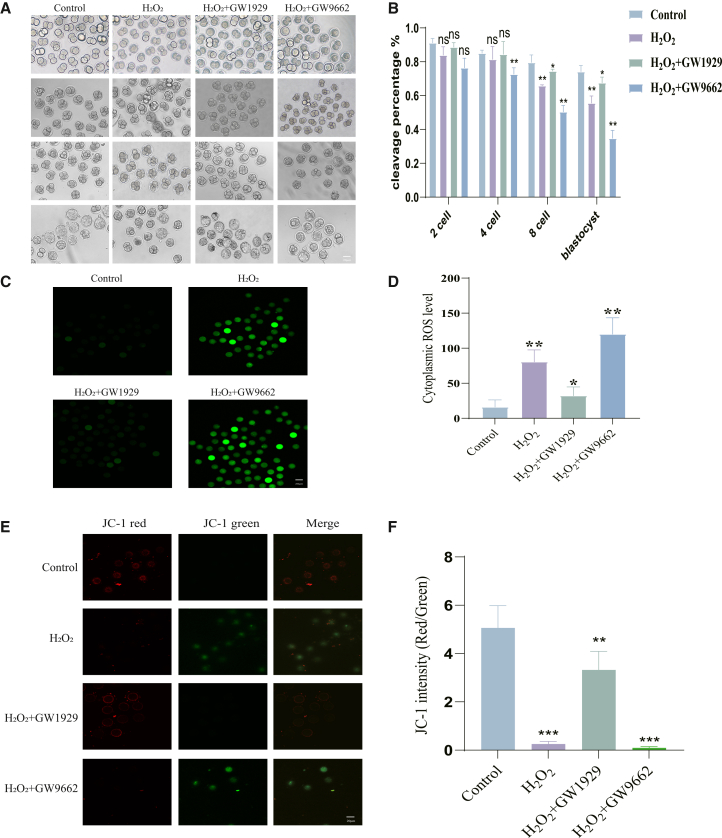
Pharmacological activation of PPARγ rescues embryonic development and alleviates oxidative stress and mitochondrial dysfunction (A) Representative bright-field images showing embryonic development to the blastocyst stage under the indicated conditions. (B) Quantification of blastocyst formation rates. (C) Representative fluorescence images of intracellular ROS levels (DHE staining, green) in zygotes. (D) Quantification of ROS fluorescence intensity from (C). (E) Representative images of mitochondrial membrane potential (JC-1 staining) in zygotes. Red fluorescence indicates high MMP (aggregates), green indicates low MMP (monomers). (F) Quantification of the red/green fluorescence ratio from (E). Data are represented as mean ± SEM; *n* > 200 per sample for development counting, n >5 per group for immunofluorescence). All data from 3 independent biological replicates. ns *p* ≥ 0.05, ∗ *p* < 0.05, ∗∗ *p* < 0.01, ∗∗∗ *p* < 0.001 by one-way ANOVA with post-hoc test. Scale bar = 20 μm.

Consistent with our hypothesis, treatment with the agonist GW1929 significantly improved the blastocyst formation rate compromised by H_2_O_2_, whereas the antagonist GW9662 exacerbated H_2_O_2_-induced developmental arrest (Figures 3A and 3B), establishing a causal role for PPARγ in promoting developmental competence.

We then elucidated the cellular mechanisms underlying this protection. First, we assessed oxidative stress and mitochondrial function. Intracellular ROS levels, which were elevated by H_2_O_2_, were effectively reduced by GW1929 and further increased by GW9662 (Figures 3C and 3D). Mitochondrial membrane potential (MMP), a key indicator of mitochondrial health that was severely impaired by oxidative stress, was significantly preserved by GW1929 and worsened by GW9662 (Figures 3E and 3F).

**Figure 4:**
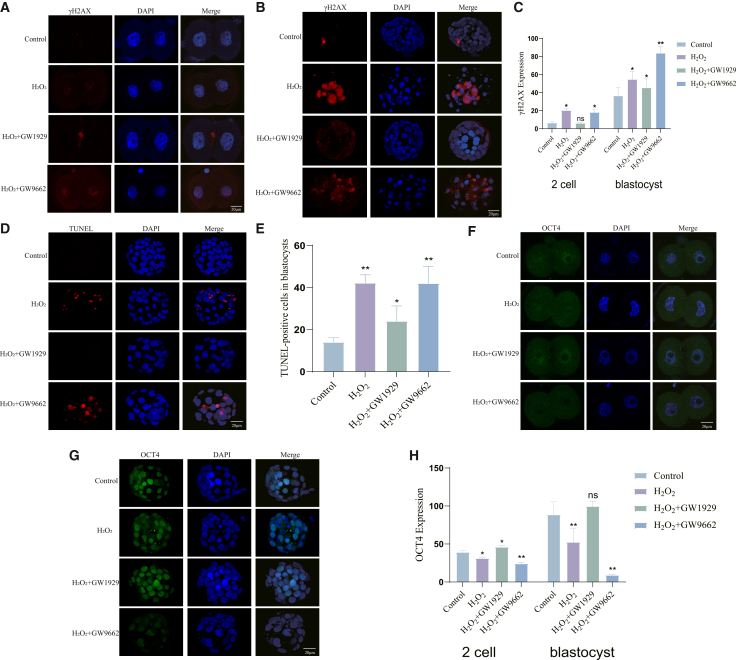
PPARγ activation attenuates oxidative damage-induced DNA damage and apoptosis, and supports pluripotency (A) Representative fluorescence micrographs of γH2AX (red) in the 2 cell embryos (control, H_2_O_2_-treated, H_2_O_2_+GW1929, and H_2_O_2_+GW9662 groups). (B) Representative fluorescence micrographs of γH2AX (red) in the blastocyst. (C) Quantification of the number of γH2AX foci per nucleus in blastocysts from (A; B). (D) Representative images of TUNEL assay (red) detecting apoptotic cells in blastocysts. Nuclei are stained with DAPI (blue). (E) Quantification of the percentage of TUNEL-positive cells per blastocyst from (D). (F) Representative immunofluorescence images of OCT4 expression (green, pluripotency marker) in 2 cell embryos. (G) Representative fluorescence micrographs of OCT4 (green) in the blastocyst. (H) Quantification of OCT4 fluorescence intensity per nucleus from (F, G). Data are represented as mean ± SEM; *n* >5 per group). All data from 3 independent biological replicates. ns *p* ≥ 0.05, ∗ *p* < 0.05, ∗∗ *p* < 0.01, ∗∗∗ *p* < 0.001 by one-way ANOVA with post-hoc test. Scale bar = 20 μm.

Furthermore, PPARγ activation safeguarded genomic integrity and pluripotency. It markedly reduced the abundance of γH2AX foci (indicative of DNA double-strand breaks) and TUNEL-positive signals (indicative of apoptosis) in blastocysts, while its inhibition had the opposite effect (Figures 4A–4E). Concomitantly, the expression of the core pluripotency marker OCT4, which is critical for embryonic development, was restored by GW1929 and further suppressed by GW9662 (Figures 4F–4H).

**Figure 5:**
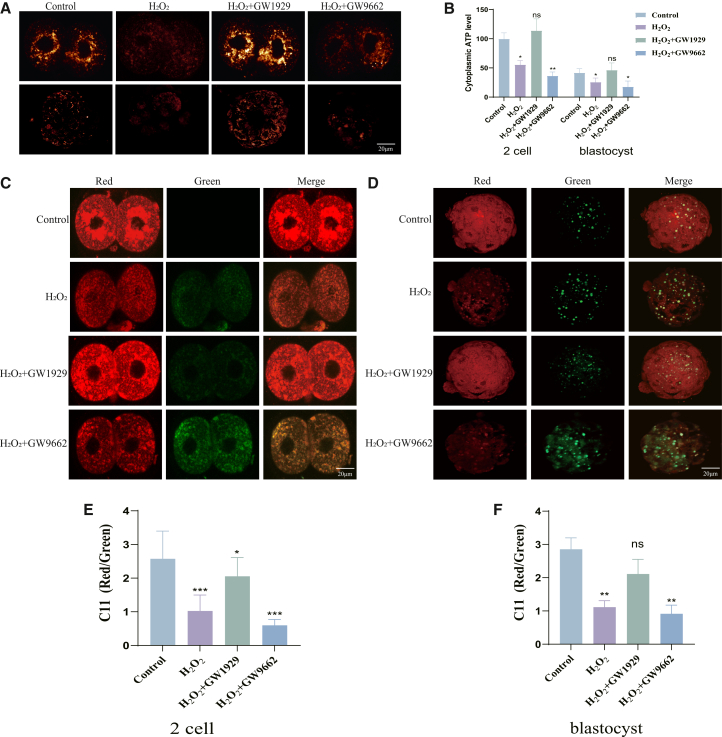
PPARγ activation maintains metabolic homeostasis under oxidative stress (A) Measurement of mitochondrial ATP levels in 2-cell and blastocyst embryos. (B) Quantification of ATP content from (A). (C) Representative images of lipid peroxidation (C11 BODIPY 581/591 staining) in 2-cell embryos. Green fluorescence indicates oxidized lipid, red indicates non-oxidized lipid. (D) Representative images of lipid peroxidation in blastocysts. (E) Quantification of the green/red fluorescence ratio (oxidized/non-oxidized) in 2-cell embryos from (C). (F) Quantification of the green/red fluorescence ratio in blastocysts from (D). Data are represented as mean ± SEM; *n* > 5 per group). All data from 3 independent biological replicates. ns *p* ≥ 0.05, ∗ *p* < 0.05, ∗∗ *p* < 0.01, ∗∗∗ *p* < 0.001 by one-way ANOVA with post-hoc test. Scale bar = 20 μm.

Notably, GW1929 treatment itself did not increase apoptosis or ROS levels above control values; it not only rescued developmental arrest but also reduced apoptotic cell ratio (Figures 4D–4E) and preserved mitochondrial membrane potential (Figures 3D–3E), excluding potential cytotoxicity of the agonist. Meanwhile, GW9662, despite exacerbating oxidative stress-induced developmental defects, did not further increase apoptosis, ROS accumulation, or lipid peroxidation (Figures 3B, 3C; 4D, 4E; 5C–5F), indicating no additional toxic effects of the antagonist at the used concentration.

Collectively, these data demonstrate that PPARγ activation protects embryos from oxidative-stress-induced damage by scavenging excessive ROS, preserving mitochondrial function, mitigating DNA damage and apoptosis, and supporting pluripotency.

### Peroxisome proliferator-activated receptor gamma maintains metabolic homeostasis under oxidative stress

Given the critical role of PPARγ in cellular metabolism and our observed defects in mitochondrial function (Figures 3E and 3F), we hypothesized that its protective role might be mediated through maintaining metabolic homeostasis. Oxidative damage led to a significant decrease in mitochondrial ATP production. This energy deficit was rescued by GW1929 treatment and exacerbated by GW9662 (Figures 5A and 5B). Furthermore, immunofluorescence analysis for lipid peroxidation suppressed oxidative stress-induced lipid damage, whereas GW9662 promoted it in both 2-cell embryos and blastocysts (Figures 5C–5F). Our initial Drug-seq data indicated alterations in the Wnt signaling pathway upon oxidative damage (Figures 1C and 1D). Given the established crosstalk between PPARγ and developmental signaling pathways such as Wnt, we explored whether this interaction also mediates the metabolic and protective effects of PPARγ during embryonic development under oxidative stress.

### Peroxisome proliferator-activated receptor gamma regulates WNT/β-catenin signaling via GSK3β

**Figure 6:**
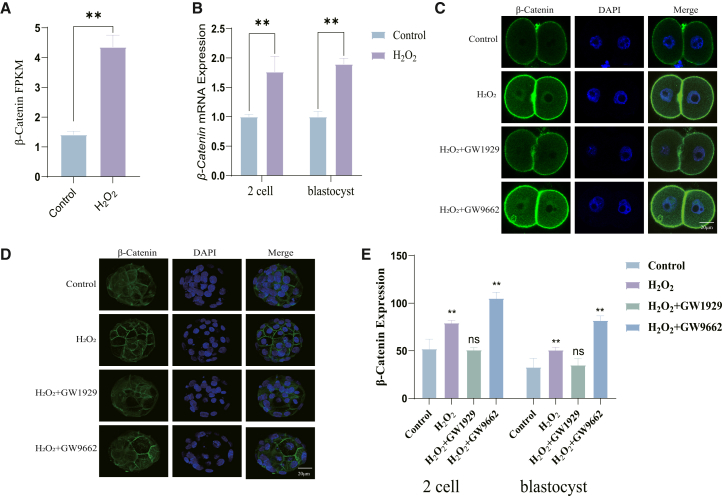
PPARγ activation suppresses oxidative stress-induced accumulation of β-catenin (A) Drug-seq results of β-catenin in control and H_2_O_2_-treated embryos at the late 2-cell stage. (B) qPCR results of β-catenin from 2 cells and blastocysts between the control vs. H_2_O_2_ groups. (C) Representative immunofluorescence images of β-catenin (green) in late 2-cell stage embryos treated with GW1929 or GW9662 after H_2_O_2_ exposure. (D) Representative images of β-catenin in blastocysts under the same conditions. (E) Quantification of β-catenin fluorescence intensity in blastocysts from (C, D). Data are represented as mean ± SEM; *n* > 5 per group for immunofluorescence, n > 150 embryos per group for PCR). All data from 3 independent biological replicates. ns *p* ≥ 0.05, ∗ *p* < 0.05, ∗∗ *p* < 0.01, ∗∗∗ *p* < 0.001 by one-way ANOVA with post-hoc test. Scale bar = 20 μm.

Analysis of our Drug-seq data indicated a significant upregulation of Ctnnb1 (β-catenin) in H_2_O_2_-treated embryos (Figure 6A). Given the known crosstalk between PPARγ and Wnt/β-catenin signaling, we investigated whether this interaction mediates PPARγ′s effects. qRT-PCR and immunofluorescence confirmed increased β-catenin expression upon oxidative damage, which was negatively regulated by PPARγ activation with GW1929 (Figures 6B–6E).

**Figure 7:**
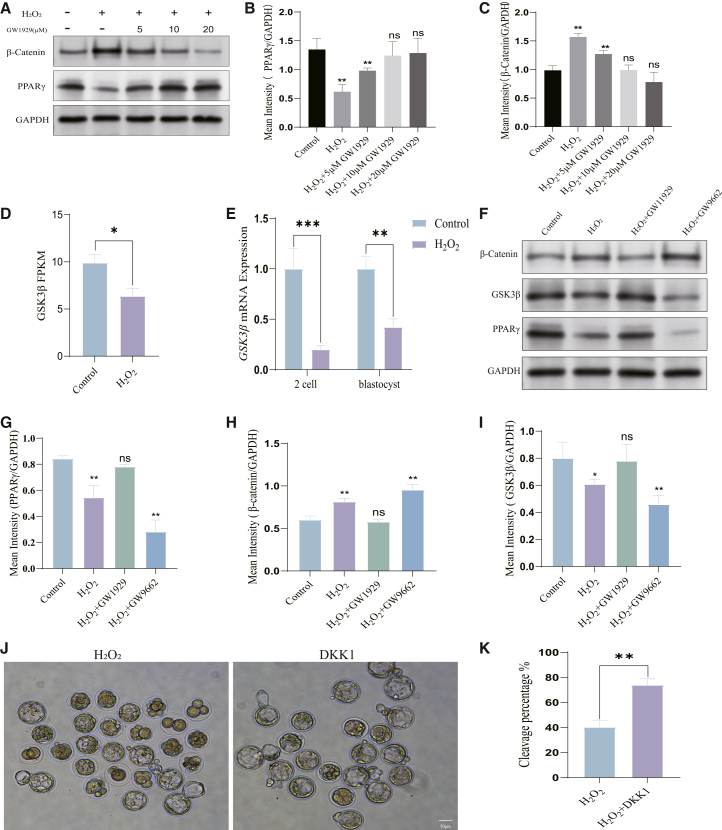
PPARγ upregulates GSK3β to inhibit β-catenin, and direct Wnt pathway inhibition mimics the protective effect of PPARγ activation (A) Western blot analysis of PPARγ, β-catenin, and GSK3β protein levels in blastocysts treated with increasing concentrations of GW1929. (B, C) Quantification of β-catenin and GSK3β protein levels from (A). (D) Drug-seq results of CTNNB1 in control and H_2_O_2_-treated embryos at the late 2-cell stage. (E) qRT-PCR analysis of GSK3β mRNA levels at different stages in control and H_2_O_2_-treated embryos. (F) Western blot analysis of β-catenin and GSK3β protein levels in blastocysts under different treatments. (G–I) Quantification of protein levels from (F). (J) Representative bright-field images showing blastocyst development after treatment with the canonical Wnt inhibitor DKK1 following H_2_O_2_ exposure. (K) Quantification of blastocyst formation rates from (J). Data are represented as mean ± SEM; *n* > 300 per group for Western blot, n > 150 per group for PCR, n > 200 per group for development counting). All data from 3 independent bilolgical replicates. ns *p* ≥ 0.05, ∗ *p* < 0.05, ∗∗ *p* < 0.01, ∗∗∗ *p* < 0.001 by Student’s *t* test (or one-way ANOVA with post-hoc test). Scale bar = 50 μm.

We next explored the mechanism by which PPARγ suppresses β-catenin. Concentration-dependent treatment with GW1929 showed that PPARγ activation led to a dose-dependent decrease in β-catenin and a concomitant increase in the expression of GSK3β (Figures 7A–7C), a kinase that promotes β-catenin degradation. GSK3β expression was itself downregulated in damaged embryos and showed a positive correlation with PPARγ activity (Figures 7D and 7E). Western blot analysis further confirmed that PPARγ activation suppressed β-catenin protein levels and enhanced GSK3β expression (Figures 7F–7I).

Crucially, the recovery of OCT4 expression following GW1929 treatment (Figures 4F–4H) suggests that the PPARγ-mediated regulation of the GSK3β/β-catenin axis is functionally linked to the maintenance of pluripotency. Collectively, these results delineate a mechanistic pathway whereby PPARγ activation upregulates GSK3β, leading to the inhibition of the aberrant WNT/β-catenin signaling induced by oxidative stress. This ultimately mitigates the downstream detrimental effects on genome integrity, apoptosis, and pluripotency, thereby promoting normal embryonic development.

Based on these findings, we propose a comprehensive model wherein oxidative stress during ZGA suppresses PPARγ, leading to the downregulation of GSK3β and consequent hyperactivation of the Wnt/β-catenin pathway. This results in metabolic dysfunction, DNA damage, and loss of pluripotency, ultimately causing developmental arrest. Conversely, PPARγ activation by GW1929 rescues this cascade by upregulating GSK3β, thereby inhibiting β-catenin and preserving embryonic viability.

To functionally test whether the inhibition of Wnt/β-catenin signaling is a key mechanism underlying PPARγ-mediated protection, we asked if direct pathway inhibition could mimic the effect of PPARγ activation. Consistent with our previous finding that Wnt pathway activation mediates long-term effects of zygotic oxidative stress,^7^ treatment with the canonical Wnt inhibitor DKK1 significantly rescued the blastocyst formation rate impaired by H_2_O_2_ (Figures 7J and 7K). This result demonstrates that the direct inhibition of Wnt/β-catenin signaling is sufficient to recapitulate the protective effect of PPARγ agonism, lending strong functional support to the proposed PPARγ-GSK3β-β-catenin axis. Together, these data demonstrate that the protective effect of PPARγ activation can be functionally mimicked by direct Wnt pathway inhibition, placing the suppression of Wnt/β-catenin signaling downstream of PPARγ.

## Discussion

The suboptimal efficiency of ART remains a significant clinical challenge. A primary etiological factor is the vulnerability of preimplantation embryos to oxidative stress induced by non-physiological *in vitro* conditions, against which their intrinsic defense mechanisms are often inadequate.^4^^,^^5^^,^^8^^,^^16^^,^^17^^,^^18^ However, the key transcriptional regulators that orchestrate the embryonic defense against such oxidative insults, particularly during the critical window of zygotic genome activation (ZGA), have remained incompletely understood.^19^ Here, we identify the nuclear receptor PPARγ as one such central guardian. We demonstrate that its essential developmental upregulation during ZGA is specifically suppressed by oxidative stress. Pharmacological activation of PPARγ robustly rescues embryonic arrest by coordinating a multi-faceted protective response: scavenging excessive reactive oxygen species (ROS), restoring mitochondrial function, and maintaining metabolic homeostasis.

Our finding that PPARγ, though present in oocytes, undergoes a developmental reactivation starting from the late 2-cell stage, coinciding with ZGA. Oxidative stress specifically blunted this reactivation (Figures 1F; 2A and 2B). ZGA represents a profound metabolic and transcriptional transition where the embryo assumes developmental control.^20^ The transition from maternal-to-zygotic control involves extensive epigenetic reprogramming, which is highly susceptible to metabolic and oxidative imbalances.^21^^,^^22^ PPARγ, a master regulator of lipid and glucose metabolism, is poised to orchestrate the necessary metabolic reprogramming, likely facilitating a shift toward oxidative phosphorylation required for continued development.^23^^,^^24^^,^^25^ The failure to activate this metabolic switch under oxidative stress, as evidenced by impaired ATP production and elevated lipid peroxidation (Figure 5), directly links PPARγ dysfunction to the collapse of bioenergetic homeostasis, a known determinant of developmental competence. This aligns with previous work by Krisher and Prather,^5^ who emphasized the importance of metabolic flexibility and the Warburg effect during preimplantation stages. Additionally, our observations of rescued ATP production and suppressed lipid peroxidation are consistent with PPARγ′s known roles in promoting oxidative phosphorylation and mitigating ferroptosis, an iron-dependent cell death driven by lipid peroxides.^26^^,^^27^^,^^28^

Beyond metabolism, our work mechanistically bridges PPARγ to the canonical Wnt pathway-a fundamental developmental signaling cascade-in the context of oxidative stress. While the antagonistic relationship between PPARγ and Wnt/β-catenin signaling has been documented in other biological contexts, such as adipogenesis and cell differentiation,^9^^,^^29^ its function in preimplantation embryos was unknown. Aberrant activation of Wnt/β-catenin signaling has been implicated in embryonic dysfunction and is associated with compromised developmental outcomes in IVF models.^7^^,^^8^^,^^30^ We establish that PPARγ activation transcriptionally upregulates GSK3β (Figure 7), the key kinase within the β-catenin destruction complex.^9^^,^^12^^,^^31^^,^^32^ This upregulation leads to the suppression of oxidative stress-induced β-catenin accumulation (Figures 6; 7). The functional centrality of this axis is underscored by our finding that the direct inhibition of the Wnt pathway with DKK1 is sufficient to rescue developmental arrest (Figures 7J and 7K), phenocopying the effect of PPARγ agonism.

The PPARγ-GSK3β-β-catenin axis integrates upstream metabolic sensing with downstream cellular outcomes to preserve embryonic fitness. By restraining aberrant Wnt/β-catenin signaling, PPARγ activation concurrently mitigates two catastrophic consequences of oxidative stress: genomic instability and loss of pluripotency. The significant reduction in DNA double-strand breaks (γH2AX foci) and apoptosis (Figures 4A–4E) reflects the preservation of genomic integrity, which is paramount for an embryo with limited DNA repair capacity.^29^^,^^33^ Concomitantly, the restoration of OCT4 expression (Figures 4F–4H) signifies the rescue of the core pluripotency network. This suggests that PPARγ, through normalizing the Wnt/β-catenin pathway, maintains an epigenetic and transcriptional landscape permissive for development.^33^^,^^34^

In conclusion, our data integrate previously disparate observations into a coherent model: oxidative stress during ZGA suppresses PPARγ, leading to diminished GSK3β expression and consequent Wnt/β-catenin hyperactivation. This disrupts metabolic homeostasis, causes DNA damage, and suppresses pluripotency, ultimately culminating in developmental arrest. Moreover, our parallel work links oxidative stress to epigenetic dysregulation,^35^ raising an intriguing future question of whether PPARγ acts as a central node coordinating both the metabolic and epigenetic reprogramming necessary for embryonic stress adaptation. Our findings not only advance our fundamental understanding of embryonic defense mechanisms but also strongly propose PPARγ agonism as a translatable therapeutic strategy. Supplementing culture media with PPARγ agonists such as GW1929 could be a promising approach to enhance embryonic resilience and improve clinical outcomes in ART by simultaneously coordinating metabolic stability, redox balance, and developmental signaling. While our data strongly suggest that PPARγ activation leads to GSK3β upregulation, future studies employing chromatin immunoprecipitation and promoter-reporter assays are required to determine whether this regulation is direct.

### Limitations of the study

Some limitations of our study should be considered. First, while our acute H_2_O_2_ model recapitulates oxidative injury, chronic low-grade stress in clinical ART may differ. Future studies exploring early 3D embryo models or clinically discarded embryos. Second, due to the limited biological material available from preimplantation embryos, we could not perform definitive mechanistic assays such as chromatin immunoprecipitation (ChIP) to confirm direct PPARγ binding to the GSK3β promoter. Third, regarding translational potential, the research agonist GW1929 provides a proof-of-concept. Subsequent work should evaluate clinically safer PPARγ modulators with established human safety data. Collectively, despite these limitations, our work elucidates a critical interaction: PPARγ activation upregulates GSK3β to antagonize Wnt/β-catenin signaling induced by oxidative stress. This defines a therapeutically targetable axis for bolstering embryonic fitness in ART.

## Resource availability

### Lead contact

Further information and requests for resources and reagents should bedirected to and will be fulfilled by the lead contact, Zhiling Li (stlizhiling@126.com).

### Materials availability

This study did not generate new unique reagents.

### Data and code availability

• Data: The raw sequence data reported in this article have been deposited in the Genome Sequence Archive (Genomics, Proteomics & Bioinformatics 2025) in the National Genomics Data Center (Nucleic Acids Res 2025), China National Center for Bioinformation/Beijing Institute of Genomics, Chinese Academy of Sciences (GSA: CRA031129) that are publicly accessible at https://ngdc.cncb.ac.cn/gsa.
• Code: This study did not generate original code. All software and computational tools used for data processing and analysis are cited in the method details section.
• Other items: Any additional information required to reanalyze the data reported in this article are available from the lead contact upon request.

## Acknowledgments

This study was supported by the 10.13039/501100001809National Natural Science Foundation of China (NSFC81871223); The Key Program of Joint Funds of the National Natural Science Foundation of China (U24A20781); The 10.13039/501100012166National Key Research and Development Program of China (2023YFC2705403); The Major Research Program of National Natural Science Foundation of China (NSFC 32370914, 92478122, 92357306). The funding sources do not play any role in interpreting the results or inferring the conclusions.

## Author contributions

L. L. and S. H. are the co-first authors. Z. L. and M. L., and H. C. are the co-corresponding authors. L. L. wrote the main article, L. L. and S. H, prepared all data and images. Z. L. and M. L., H. C. revised the final article. All authors read and approved the final article.

## Declaration of interests

The authors declare no competing interests.

## STAR★Methods

### Key resources table

**Table:** 

REAGENT or RESOURCE	SOURCE	IDENTIFIER
**Antibodies**		

PPARγ (Rabbit monoclonal, clone C26H12)	Cell Signaling Technology	Cat# 2435S; RRID: AB_2166051
β-Catenin (Rabbit monoclonal, clone D10A8)	Cell Signaling Technology	Cat# 8480S; RRID: AB_11127855
GSK3β (Rabbit monoclonal, clone 27C10)	Cell Signaling Technology	Cat# 12456S; RRID: AB_2636978
OCT4 (Rabbit monoclonal, clone C30A3)	Cell Signaling Technology	Cat# 75463S; RRID: AB_2924513
γH2AX (Mouse monoclonal, clone 2F3)	Santa Cruz Biotechnology	Cat# sc-517348; RRID: AB_2924514
HRP-conjugated anti-Rabbit IgG (Goat)	Cell Signaling Technology	Cat# 7074S; RRID: AB_2099233
Alexa Fluor® 488 anti-Rabbit IgG (Goat)	Abcam	Cat# ab150077; RRID: AB_2630356
Alexa Fluor® 594 anti-Mouse IgG (Goat)	Abcam	Cat# ab150116; RRID: AB_2650601

**Chemicals, peptides, and recombinant proteins**		

GW1929 (PPARγ agonist)	MedChemExpress	Cat# HY-15655; CAS: 222919-55-9
GW9662 (PPARγ antagonist)	MedChemExpress	Cat# HY-16578; CAS: 22978-25-2
Recombinant Mouse DKK1 Protein	R&D Systems	Cat# 5439-DK-010
Hydrogen Peroxide (H_2_O_2_)	Sigma-Aldrich	Cat# H1009; CAS: 7722-84-1
Pregnant Mare Serum Gonadotropin (PMSG)	Ningbo Second Hormone Factory	N/A
Human Chorionic Gonadotropin (hCG)	Ningbo Second Hormone Factory	N/A
Bovine Serum Albumin (BSA), Fraction V	Sigma-Aldrich	Cat# A1933
Dihydroethidium (DHE)	Beyotime	Cat# S0033
JC-1 Mitochondrial	Beyotime	Cat# C2006
ATP Red-1	MedChemExpress	Cat# HY-U00451
C11-BODIPY (581/591)	GLPBIO	Cat# GC40165; CAS: 1280132-27-4
Tyrode’s Acid Solution	Sigma-Aldrich	Cat# T1788

**Critical commercial assays**		

RNeasy Mini Kit	Qiagen	Cat# 74106
RNAprep Pure Micro Kit	Tiangen Biotech	Cat# DP420
FastKing gDNA Dispelling RT SuperMix	Tiangen Biotech	Cat# KR128
TB Green Premix Ex Taq™ II (Tli RNaseH Plus)	Takara	Cat# RR420Q
riboAPO™ One-Step TUNEL Apoptosis Detection Kit (Red Fluorescence)	Ribobio	Cat# C11026-1

**Deposited data**		

Raw and analyzed DRUG-seq data	This paper	GSA: CRA031129

**Experimental models: Organisms/strains**		

Mouse: C57BL/6	Beijing Vital River Laboratory Animal Technology Co., Ltd.	RRID: IMSR_JAX:000664

**Oligonucleotides**		

Primers for qPCR, see Table S1	Sangon Biotech	N/A

### Experimental model and study participant details

This study did not involve human participants. All experiments used a mouse model.

#### Mouse model (C57BL/6J)

##### Source

Beijing Vital River Laboratory Animal Technology Co., Ltd.

##### Age

Female mice (6-8 weeks old) were used for oocyte retrieval, and male mice (3-6 months old) served as sperm donors. Both sexes functioned exclusively as gamete providers.

##### Sex

In this study, preimplantation embryos were obtained via IVF. The sex of the resulting embryos was not determined, as all experiments were performed at the preimplantation stage, prior to the onset of sex differentiation.

##### Housing and husbandry

Mice were housed in a specific pathogen-free (SPF) barrier facility under a 12-h light/dark cycle at 20-26°C and 40-70% humidity. Food and water were provided *ad libitum*.

##### Allocation to experimental groups

Oocytes and sperm were pooled from multiple donors to reduce individual variability. Zygotes derived from these pooled gametes were randomly assigned to control or treatment groups, which effectively minimized potential litter-of-origin confounding effects.

##### Ethics statement

All animal experimental protocols were approved by the Laboratory Animal Ethics Committee of Shantou University Medical College (Approval Number: SUMC2021-502). All procedures were performed in strict compliance with the International Guidelines for Biomedical Research Involving Animals (Council for International Organizations of Medical Sciences [CIOMS], 2012).

#### Preimplantation embryos (derived from C57BL/6 mice)

##### Source

Generated by *in vitro* fertilization (IVF) using gametes from the mice described above.

##### Culture conditions

Embryos were cultured in HTF medium (SAGE, ART-1020) supplemented with 0.4% BSA (Sigma, A1933) at 37°C under 5% CO_2_.

##### Sex of embryonic subjects

The sex of the embryos was not determined, as all experiments were performed at the preimplantation stage, prior to the onset of gonadal sex differentiation. Consequently, any potential influence of embryo sex on the reported results was not assessed.

##### Developmental stages of subjects

The primary subjects of this study were preimplantation mouse embryos. Embryos at specific developmental stages were collected for analysis: 2-cell early embryos (20-22 hours post-insemination, hpi), 2-cell late embryos (28-30 hpi), and blastocyst (96 hpi) stages. The collection timeline relative to treatments is detailed in Figure lemen, A) Preimplantation embryo collection at different stages.

### Method details

#### Superovulation and gamete collection

Female mice were superovulated via intraperitoneal injection of 10 IU pregnant mare serum gonadotropin (PMSG; Ningbo Second Hormone Factory), followed by administration of 10 IU human chorionic gonadotropin (hCG; Ningbo Second Hormone Factory) 48 h later. Cumulus-oocyte complexes (COCs) were harvested from the oviducts at 13-15 h post-hCG injection.

#### Sperm collection and capacitation

Sperm were collected from the epididymis and vas deferens of male mice and capacitation in HTF medium (AibeiBio, Cat. No. M2050) and capacitated for 1 h at 37°C in a humidified atmosphere of 5% CO_2_.

#### *In vitro* fertilization (IVF) and embryo culture

COCs were co-incubated with capacitated spermatozoa for 4-6 h. Following thorough washing, zygotes were cultured in fresh HTF medium supplemented with 0.4% bovine serum albumin (BSA), under humidified conditions of 37°C and 5% CO_2_.

#### Oxidative stress

H_2_O_2_-induced oxidative stress: Zygotes were exposed to 0.03 mM hydrogen peroxide (H_2_O_2_; Sigma-Aldrich, Cat. No. H1009) for 30 min to induce oxidative stress.

#### PPARγ agonism and inhibition

Immediately following H_2_O_2_ exposure, embryos were treated with 10 μM GW1929 (PPARγ agonist; MCE, Cat. No. HY-15655)^36^ or 1.25 μM GW9662 (PPARγ antagonist; MCE, Cat. No. HY-16578)^37^ for 1 h, and maintained in the same treatment medium thereafter.

#### Wnt pathway inhibition

In parallel experiments, after H_2_O_2_ treatment, embryos were cultured in medium containing 1000 ng/ml recombinant Dickkopf-related protein 1 (DKK1; R&D Systems, Cat. No. 5439-DK) for 1 h. Subsequently, embryos were washed three times with fresh culture medium and continued to be cultured under standard conditions.

#### EpiTM DRUG-seq analysis

Total RNA was extracted from early 2-cell and late 2-cell stage embryos using the RNeasy Mini Kit (Qiagen, 74106). Library preparation and sequencing were performed following the manufacturer’s instructions. Raw sequencing reads were quality-filtered and aligned to the mouse reference genome (GRCm39) using Bowtie2. Gene expression levels were quantified as FPKM. Differential expression analysis was conducted using DESeq2 with thresholds of |log_2_FC| ≥ 2 and adjusted p-value ≤ 0.05.

#### Quantitative real-time PCR (qRT-PCR)

Total RNA was extracted from 150 embryos using the RNAprep Pure Micro Kit (Tiangen, DP420). cDNA was synthesized with FastKing gDNA Dispelling RT SuperMix (Tiangen, KR128). qPCR was performed using TB Green Premix Ex Taq™ (Takara, RR420Q) on a CFX96 Real-Time PCR System (Bio-Rad). GAPDH served as the internal control. Relative gene expression was calculated using the 2−ΔΔCt method. The primers are shown in Table S1. Primers used for qRT-PCR. A) Forward and reverse primer sequences for each target gene.

#### Western blot

Protein lysates were extracted from 300 embryos using RIPA buffer supplemented with protease and phosphatase inhibitors (Yeasen, 20101). Proteins were separated by SDS-PAGE, transferred to PVDF membranes, and probed with primary antibodies (PPARγ antibody (1:1000; CST, 2435S), β-Catenin antibody (1:1000; CST, 8480S), GSK3β antibody (1:1000; CST, 12456) overnight at 4°C. After incubation with HRP-conjugated secondary antibodies (1:1000; CST, 7074S), bands were visualized using chemiluminescence and quantified with BandScan 5.0.

#### Immunofluorescence staining

Zonae pellucidae were removed using Tyrode’s acid solution (Sigma, T1788). Embryos were fixed in 4% PFA for 30 min, permeabilized with 0.5% Triton X-100, and blocked in PBS containing 3% BSA and 10% goat serum. Primary antibodies (PPARγ antibody (1:200; CST, 2435S), β-Catenin antibody (1:200; CST, 8480S), OCT4 (1:200; CST, 75463S), γH2AX (1:200; Santa, sc-517348) were applied overnight at 4°C, followed by Alexa Fluor-conjugated secondary antibodies (1:200, ab6717 and ab150115, Abcam) and DAPI (Solarbio, C0060) counterstaining.

Images were acquired using a confocal microscope (Nikon) and analyzed with ImageJ.

#### TdT-mediated dUTP nick-end labeling (TUNEL) assay

Apoptosis was detected using the riboAPO™ One-Step TUNEL Apoptosis Detection Kit (Ribobio, C11026-1). Blastocysts were fixed and permeabilized as above, incubated with TUNEL reaction mixture for 1 h at 37°C, and counterstained with DAPI. The apoptosis rate was calculated as the percentage of TUNEL-positive cells per total cells. This assay was used to quantify apoptotic cells, a direct indicator of drug-induced cytotoxicity. By calculating the percentage of TUNEL-positive cells, we evaluated whether GW1929 or GW9662 caused abnormal cell death beyond oxidative stress-induced damage.

#### Measurements of ROS, MMP, ATP, and lipid peroxidation

ROS: Embryos were incubated with 10 μM DHE (Beyotime, S0033) for 20 min and imaged under a fluorescence microscope.

MMP: JC-1 staining (Beyotime, C2006) was performed according to the manufacturer’s instructions.

ATP: ATP levels were measured using ATP Red-1 (MCE, HY-U00451).

Lipid peroxidation: C11 BODIPY 581/591 (GLPBIO, GC40165) was used to assess lipid ROS.

ROS and lipid peroxidation levels were measured to assess oxidative cytotoxicity, as excessive ROS accumulation and lipid peroxidation are key mechanisms of drug-induced cell damage. MMP was detected to evaluate mitochondrial integrity, an early marker of cytotoxicity-related mitochondrial dysfunction. These assays not only reflect the potential toxic effects of GW1929 and GW9662 on embryonic cells but also effectively evaluate the protective role of PPARγ in oxidatively damaged embryos.

#### Experimental design and rigor

##### Replication

All experiments were conducted with at least three independent biological replicates, defined as separate IVF sessions or embryo cohorts.

##### Randomization

Zygotes derived from pooled gametes were randomly allocated to different treatment groups to ensure unbiased distribution.

##### Blinding

For endpoint analyses requiring manual counting (e.g., blastocyst rate, TUNEL-positive cells) or image quantification, the experimenter was blinded to group identities during data collection and analysis whenever feasible.

##### Sample size

Sample sizes (number of embryos per group) were determined based on preliminary data and follow standard practices in preimplantation embryology to ensure sufficient statistical power. Exact n values for each experiment are reported in the figure legends.

##### Inclusion/exclusion criteria

Only morphologically normal embryos at the appropriate developmental stage (evaluated via bright-field microscopy) were included in subsequent analyses.

### Quantification and statistical analysis

#### Statistical tests

Data were analyzed using SPSS 19.0 and GraphPad Prism 8.4.3. Comparisons between two groups were performed using two-tailed Student’s t-test. Comparisons among three or more groups were performed using one-way ANOVA followed by Tukey's post-hoc test for multiple comparisons. Categorical data (blastocyst formation rates) were analyzed using Chi-square test.

#### Definition of n

The “n” reported in figure legends represents the number of independent biological replicates (experimental repeats) unless otherwise stated. For assays on individual embryos (e.g., fluorescence intensity), the total number of embryos analyzed across all replicates is also provided.

#### Data presentation

Data are represented as mean ± SEM. Individual data points are shown in graphs where applicable.

#### Significance threshold

A p-value < 0.05 was considered statistically significant. Exact p-values are reported in the figures or figure legends as follows: ns *p* > 0.05, ∗ *p* < 0.05, ∗∗ *p* < 0.01, ∗∗∗ *p* < 0.001.

#### Statistical details

The specific statistical test used, exact n numbers, and p-values for each experiment are detailed in the corresponding figure legends.

#### Additional resources

This study did not generate new standalone websites, databases, or clinical trial registrations. All primary data have been deposited in a public repository as stated below.

## Supplemental Information

Document S1. Figure S1

Table S1. Primers used for quantitative real-time PCR in this study. A.) Forward and reverse primer sequences for each target gene
