# Supplementary material for: PPARγ activation rescues oxidative stress-induced embryonic arrest by suppressing Wnt/β-catenin signaling via GSK3β upregulation
Source: iScience. 2026 Jan 30;29(3):114870. doi: 10.1016/j.isci.2026.114870 (PMC12924746; doi:10.1016/j.isci.2026.114870)
Supplement: Document S1. Figure S1 [file mmc1.pdf]

iScience, Volume *29*

Supplemental information

PPARγ activation rescues oxidative stress-induced embryonic arrest by suppressing Wnt/β-catenin signaling via GSK3β upregulation

Lihong Liu, Siyao Ha, Hui Chen, MingQing Li, and Zhiling Li

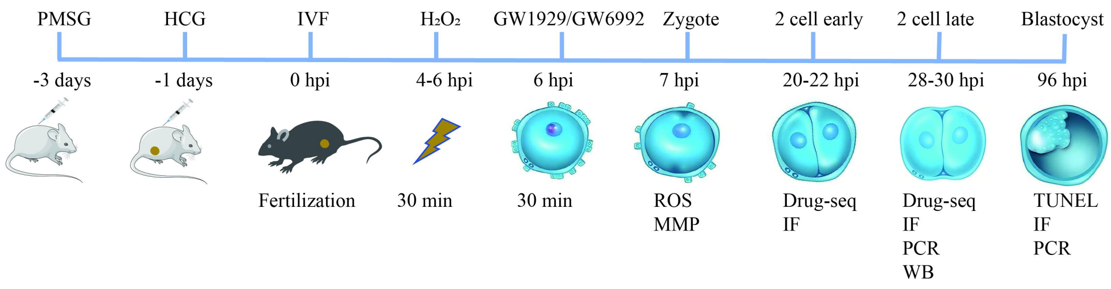

Figure S1. Fertilization process and cell collection time points across developmental stages.
